# Durable clinical benefit of letrozole in leptomeningeal metastasis of breast cancer

**DOI:** 10.1007/s13691-019-00372-8

**Published:** 2019-04-19

**Authors:** Toshihiro Takanashi, Hajime Hikino, Yoshinari Makino, Yoko Murata

**Affiliations:** Department of Breast Surgery, Matsue Red Cross Hospital, 200 Horo, Matsue, Shimane 690-8506 Japan

**Keywords:** Leptomeningeal metastasis, Breast cancer, Hormone therapy

## Abstract

A case of a woman in her 60s with breast cancer, whose leptomeningeal metastasis (LM) of breast cancer improved remarkably with letrozole monotherapy, is reported. The patient complained of numbness of her left hand and hoarseness, followed by progressive asymmetric extremity weakness and a bladder and rectal disturbance. The patient had undergone surgery for left breast cancer 18 years earlier and was concerned about recurrence of breast cancer, but there were no typical findings with some imaging modalities. The third lumbar puncture showed the malignant cytology of breast cancer, and the patient was diagnosed with recurrent breast cancer. Her performance status was very poor, and it was difficult to administer systemic chemotherapy. Letrozole was started because immunohistochemistry was positive for estrogen and progesterone receptors. After 4 months of letrozole therapy, the symptoms improved gradually. LM has a poor prognosis, and there is little evidence on which to base treatment, but hormone therapy may be an option for LM when the tumor is hormone receptor-positive, slow growing, and has a small volume.

## Introduction

Leptomeningeal metastasis (LM) of breast cancer is a relatively rare form of metastatic breast cancer that metastasizes to the meninges. Its prognosis is very poor, and it is one of the most challenging conditions to diagnose and treat, because the symptoms of LM are often similar to those of neurologic or orthopedic disorders. The patients’ performance status is often poor, and there is little evidence on which to base the treatment of LM with the interventions that have been tried to date. A systematic review showed that the median survival of patients with LM was only 15 weeks [1]. A case of LM from breast cancer with long-term survival and stabilization with letrozole monotherapy is reported.

## Case report

**Fig. 1:**
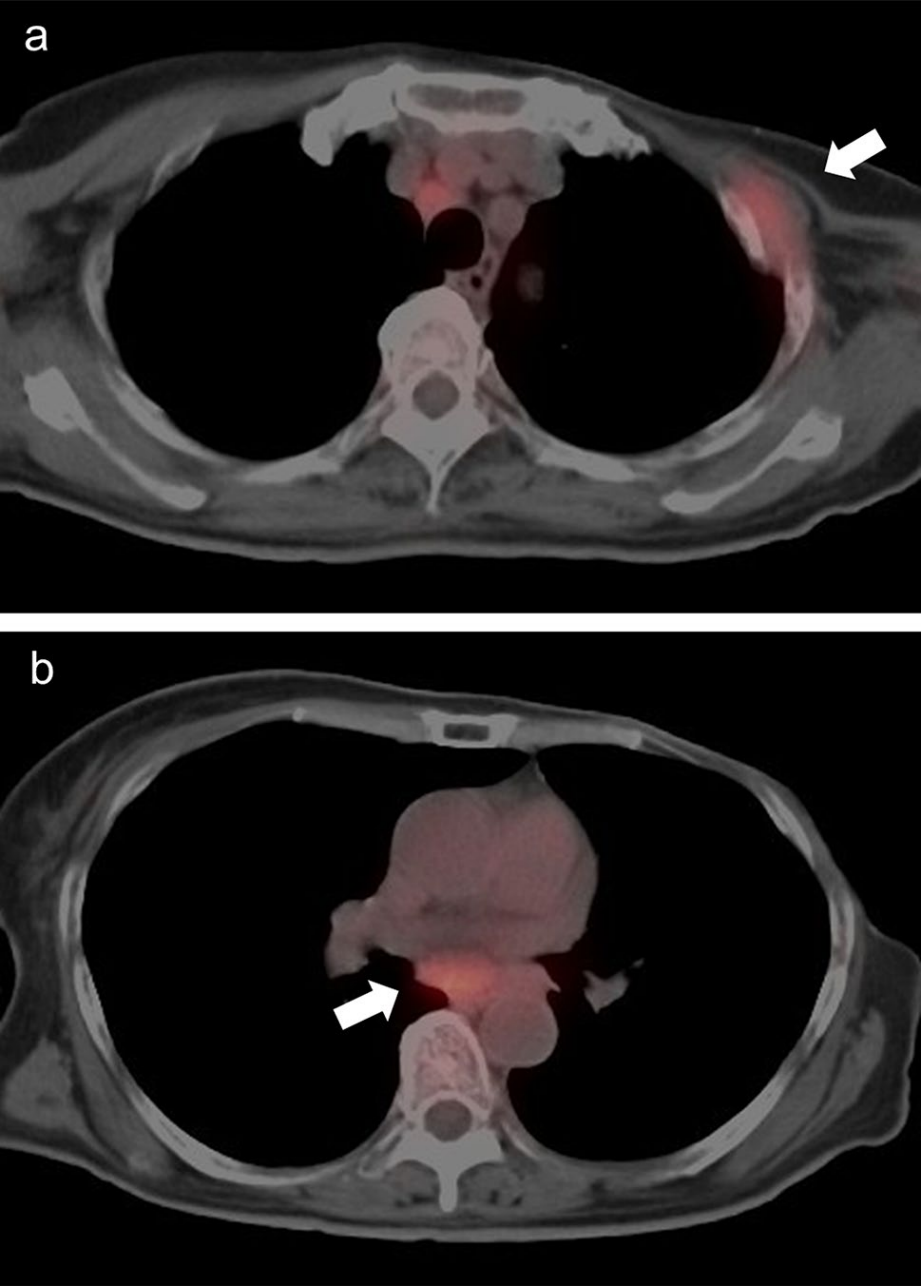
PET-CT shows abnormal uptakes in the left thoracic wall (**a**) and mediastinum (**b**) that indicate local recurrence at the thoracic wall and lymph node metastasis (see arrows)

**Fig. 2:**
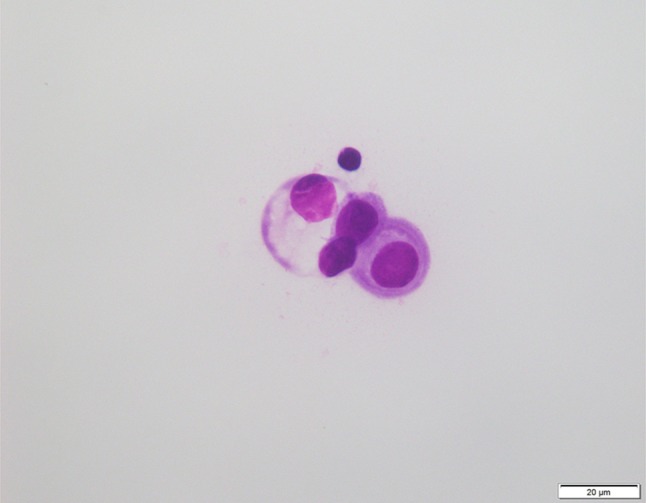
Atypical cells in Giemsa staining on CSF cytology. Morphologic features showed an increased nuclear–cytoplasmic ratio and various sizes of nuclei, which indicate leptomeningeal metastasis of breast cancer

A woman in her 60s had a mastectomy for left breast cancer 18 years earlier. In February 2014, she developed numbness of her left hand and hoarseness, followed by progressive asymmetric extremity weakness and a bladder and rectal disturbance. The neurologic symptoms were inconsistent with any neurologic or orthopedic diseases. Positron emission tomography–computed tomography was performed due to suspected relapse of breast cancer, and it showed possible local recurrence involving the left thoracic wall and metastasis to a mediastinal lymph node (Fig. 1). The biopsy of the thoracic wall region showed recurrence of breast cancer, and immunohistochemistry was positive for estrogen and progesterone receptors, but negative for tissue HER2 over-expression. Gadolinium contrast-enhanced magnetic resonance imaging (MRI) of the head and spine showed no abnormalities, and cerebrospinal fluid (CSF) analysis was negative for malignancy. Considering a paraneoplastic syndrome, the antibodies were checked, but there were no abnormalities. Although a second CSF analysis was also negative, the third analysis demonstrated malignant cells consistent with adenocarcinoma of the breast (Fig. 2). She was not treated with radiation therapy because of her poor general condition (Karnofsky’s index of performance status 30), and there was no target to irradiate. She also could not be treated with systemic chemotherapy due to her condition because of the potential side effects, and she was started on oral letrozole, which has a low incidence of side effects. After 4 months of letrozole therapy, the strength and numbness of her extremities improved gradually, and she was finally able to walk. At 11 months of letrozole therapy, CT showed reduction in size of the left thoracic wall’s recurrence and the mediastinal lymph node metastasis. There were no adverse events. Serum CA15-3 as a tumor marker was increased (30.6 U/ml) before she started hormone therapy, but it decreased after she started letrozole and then remained low. She has so far achieved long-term survival with more than 30 months with no progression of LM.

## Discussion

LM occurs clinically in about 1–2% of all breast cancer cases [2]. Most commonly, it develops in patients in the terminal stage with metastases to other organs. LM is clinically characterized by multifocal neurological signs and symptoms [3]. Spinal manifestations of LM include weakness of the extremities, a bladder and rectal disturbance, and back and neck pain, which often affect patients’ QOL. Diagnosing LM can sometimes be confusing because the symptoms are similar to those of neurologic and orthopedic diseases. With regard to the treatment of LM, standard treatment for this disease has not yet been developed, and the prognosis is still poor. Therefore, diagnosis and treatment of LM have long been big issues in breast surgery departments. Meningeal tumor deposits are well vascularized [3], and gadolinium contrast-enhanced MRI typically shows enhancement of the meninges, but findings are sometimes absent [4]. Demonstration of malignant cells in the CSF is needed to make a definitive diagnosis. Positive cytology is found in only 50% of patients with LM on the initial CSF analysis, and the probability of the diagnosis increases to 85% with three or more CSF analyses [5]. Thus, careful assessment is required to diagnose LM.

It is thought that most chemotherapy drugs do not cross the blood–brain barrier (BBB) [6], and standard treatment for LM has historically consisted of intrathecal (IT) chemotherapy. Niwińska et al. published an investigation of prognostic factors in LM, and 75% of the patients who had prolonged survival were treated with IT chemotherapy [7]. However, IT chemotherapy requires invasive procedures and has a higher treatment complication rate [8, 9], and it can often make a difficult situation worse [10]. Thus, IT chemotherapy has been administered less recently. There have been several reports of patients with LM responding to systemic chemotherapy [11, 12]. The CSF is an unfavorable environment for tumor cells; those that survive adhere to the vascularized arachnoid and grow out to form local tumor infiltrates. Meningeal tumor deposits are well vascularized with highly permeable blood vessels. Treatment for these tumor deposits by cytostatic drugs through their own blood supply may be more effective than by superficial exposure to drugs dissolved in the CSF [13].

There are several published reports of successful treatment of LM with hormone therapies such as tamoxifen, letrozole, and exemestane [2, 5, 6, 8, 14–16]. Tamoxifen is a selective estrogen receptor modulator that is a highly lipophilic agent and can presumably cross the BBB easily [7]. Miyajima et al. reported that letrozole may traverse the BBB easier than other aromatase inhibitors in the CNS of mice [17]. However, the mechanism of penetration of these drugs into the CNS remains unknown.

Most patients with LM have metastases to other organs such as liver, brain, and bone. However, the present case had only local recurrence, and MRI did not show the typical findings of LM or other metastases, which suggested that tumor volume was relatively small. In addition, the present case might have had an advantage because of the very long disease-free interval and the tumor tended to be slow growing. Hormone therapy, with its ease of use and advantageous side-effect profile, could be a first choice for the treatment for LM when the tumor is hormone receptor-positive, slow growing, and has a small volume. However, the limitation is the lack of evidence for treatment with hormone therapy for LM. There have been no randomized, prospective trials of hormone therapy for LM because the number of patients with LM is relatively small. There have been only several case reports of successful hormone therapy for LM. Recently, the potential role of cyclin-dependent kinase 4/6 inhibitors against breast cancer brain metastases is being studying in ongoing clinical trials [18]. Combination therapy of hormone and cyclin-dependent kinase 4/6 inhibitors may also be effective in the treatment for LM because of the anticipated synergistic antitumor effect. Accumulating further cases and establishing evidence for the tolerable therapy of LM would be desirable.
